# Long‐Term Outcomes After Prophylactic Infusion of 2% Tetrasodium Ethylenediaminetetraacetic Acid in 95 Subcutaneous Ureteral Bypass Devices in 66 Cats With Benign Ureteral Obstructions

**DOI:** 10.1111/jvim.70006

**Published:** 2025-02-26

**Authors:** Allyson Berent, Chick Weisse, Melissa Milligan, Jessica Mejia, Sarah Woods, Ken Lamb

**Affiliations:** ^1^ The Animal Medical Center New York New York USA; ^2^ Lamb Consulting St. Paul Minnesota USA

**Keywords:** biofilm, device, encrustation, mineralization, SUB, ureterolithiasis

## Abstract

**Background:**

The use of subcutaneous ureteral bypass device(s) (SUB) for the treatment of benign ureteral obstructions (BUO) in cats has become more routine in veterinary practices. Device mineralization and chronic urinary tract infections (UTI) are the most reported long‐term complications.

**Hypothesis/Objective:**

To evaluate the occurrence of mineralization, bacteriuria, and chronic infections in cats after SUB placement, where 2% tetrasodium ethylenediaminetetraacetic acid (tEDTA) was infused during standard SUB flushing when compared to a historical control group where sterile saline was used for flushing. The hypothesis is that tEDTA will decrease the rate of mineralization occlusions and infections of SUB devices without increasing other complications.

**Animals:**

Ninety‐five SUBs (39 unilateral; 28 bilateral) in 66 cats.

**Methods:**

Retrospective study; Medical records from all cats that had > 180‐day follow‐up after undergoing SUB device placement were evaluated. Group 1 consisted of cats flushed routinely with saline only, Group 2 with saline and then switched to 2% tEDTA during their follow‐up period, and Group 3 with 2% tEDTA only.

**Results:**

Device mineralization was documented in 9/28 (32%), 8/16 (50%), and 10/51 (19%) SUB devices in Groups 1, 2, and 3, respectively (*p* = 0.025). Exchange was needed from re‐obstruction in 4/28 (14%), 5/16 (31%) and 3/51 (6%), respectively (*p* = 0.016). Chronic UTI occurred in 7/21 (33%), 4/12 (33%), and 1/33 (3%) of cats, respectively (*p* = 0.004). UTI at the time of surgery was associated with the development of a chronic UTI (*p* = 0.01).

**Conclusions/Clinical Importance:**

Prophylactic use of 2% tEDTA might be helpful in reducing frequency of long‐term SUB device complications and could be considered as part of the long term management plan.

**Abbreviations**
ABU: asymptomatic bacteriuria
BUN: blood urea nitrogen
BUO: benign ureteral obstruction
CKD: chronic kidney disease
SUB: subcutaneous ureteral bypass device
tEDTA: 2% tetrasodium ethylenediaminetetraacetic acid
UCS: urine culture and sensitivity
UTI: urinary tract infection

## 1 Introduction

The use of a subcutaneous ureteral bypass device (SUB)^1^ for the treatment of BUOs has been reported by numerous groups, with the largest series in 134 cats showing good to excellent outcomes [1, 2, 3, 4, 5, 6, 7]. In the authors' practice, this device has essentially replaced traditional surgery and ureteral stenting for the treatment of ureteral obstructions in cats, regardless of etiology, because of lower morbidity and mortality rates [1, 2, 3, 4, 5, 6, 7, 8, 9, 10, 11, 12, 13, 14]. Over the past decade, the use of the SUB has become the standard of care for many institutions globally for the treatment of BUO [1, 2, 3, 4, 5, 6, 7, 9]. This trend is likely due to ease of placement regardless of the underlying etiology or location of the obstructive lesion, generally resulting in shorter surgical times, lower re‐obstruction rates, and fewer perioperative complications, with overall improved morbidity and mortality rates compared to traditional surgery [8, 10, 11, 12, 13, 14].

Additionally, SUBs can be successfully used for nearly all obstructive etiologies including ureterolithiasis, ureteral strictures, obstructive neoplasia, obstructive pyonephrosis, and dried solidified blood stones, where traditional surgical approaches can be incredibly challenging and might not be possible [1, 3, 6, 15]. The SUB has also generally replaced the use of ureteral stents in cats globally because of the lower incidence of stent‐associated dysuria and the ease of SUB placement [8, 10, 11, 12]. Despite the SUB improving many of the outcomes, complications such as chronic mineralization (24%), the need for device exchange (13%), and the development of chronic infections (8%) are reported [1].

After SUB placement, an ultrasound‐guided flush of the device is routinely recommended [1]. Since mid‐2017, instillation of 2 mL of 2% tEDTA^2^ solution (tEDTA) into the device has been standard in the authors' practice (Figure S1). The tEDTA is a chelating agent that complexes with cations (e.g., calcium). In the literature, irrigation with tEDTA achieved complete dissolution of stones in 145/260 cases, while partial dissolution was seen in 113 cases [16]. tEDTA‐treated‐catheters have significantly lower rates of encrustation compared to controls [17], and tEDTA can demineralize occluded SUBs [18, 19]. This has been met with success in 8/8 cases reported [18].

The objective of this study was to evaluate the incidence of mineralization, infections, dysuria, and azotemia when tEDTA was routinely infused into each SUB when compared to a saline infusion control group. The hypothesis to be tested is that tEDTA decreases the rate of mineralization and infections without worsening dysuria or azotemia in cats when compared to saline.

## 2 Materials and Methods

### 2.1 Selection of Cases

The medical records of all cats undergoing SUB placement for the treatment of BUOs by the authors (Allyson Berent or Chick Weisse, or both) between January 2012 to July 2020 treated at the Animal Medical Center, New York, New York, were retrospectively reviewed. Diagnosis of a ureteral obstruction was generally made based on a combination of imaging findings detected on abdominal ultrasonography (renal pelvic dilation with associated hydroureter usually caused by a nonmalignant obstructive ureteral lesion), abdominal radiographs, and an antegrade ureteropyelography performed at the time of SUB placement using fluoroscopic guidance. Preoperative diagnostic imaging, preoperative serum biochemistry, and a complete surgery report were needed for inclusion with a follow‐up time of > 180 days for each cat. Cats were excluded if < 180 days of follow‐up data was available for review and > 180 days lapsed between SUB flushing during the follow‐up period. If mineralization or a UTI occurred before the first 180 days but the follow‐up time was < 180 days, these cats were included. Cats and the SUB(s) were grouped based on the material used in their routine SUB flushing procedure. UTI was defined as a positive urine culture and sensitivity (UCS) with associated pyuria and clinical signs including dysuria, nonspecific lethargy, hyporexia, change in behavior, or progressive azotemia. If these criteria were not met, but the urine culture was positive the cat was classified as having asymptomatic bacteriuria (ABU). Chronic UTI was defined as having more than two episodes of UTI in the preceding 12 months or two or more episodes in the preceding 6 months [20]. Cats were considered to have successfully cleared an infection if a negative UCS was obtained while not receiving medical treatment for infection without recurrence for > 6 months. Postoperative sterile dysuria was defined as the development of any lower urinary tract signs in the absence of a positive UCS.

Group 1 included devices flushed with sterile saline only (devices placed from 4/2012 to 4/2017), Group 2 included devices initially flushed with sterile saline and then switched to 2% tEDTA (devices placed from 5/2012 to 4/2017), and Group 3 included devices only flushed with tEDTA (devices placed from 5/2017 to 7/2021). Data on all cats enrolled was collected until September 2024.

### 2.2 Preoperative Data and Management

Data regarding signalment, history, presenting complaint, imaging findings, and laboratory results were reviewed.

The cats were medically managed for variable lengths of time before SUB placement depending on clinical status. Medical management included intravenous fluid therapy, alpha‐adrenergic blockade, antibiotics, H2‐receptor antagonists or proton pump inhibitors, and antiemetics; specific drug choices and doses were clinician‐dependent. The cats were monitored by physical exam, hydration assessment, serial ultrasonography, and renal variables.

### 2.3 Imaging

Generally, all cats underwent an abdominal ultrasonography (at a minimum focused on the urinary tract) and abdominal radiographs before surgery.

Fluoroscopic anterograde ureteropyelography was performed by laparotomy to confirm the ureteral obstruction in each renal/ureteral unit before SUB placement. This was generally performed using an 18‐gauge over‐the‐needle catheter and a 50% mixture of saline and iodinated contrast material [1]. When possible, urine was collected at the time of pyelocentesis for a urine bacterial culture.

### 2.4 SUB Device Placement

The SUB was placed routinely as previously reported and further defined in the SUB instruction for use manual [1, 21].^3^ For the SUB 2.0 (Figure S2a), both the nephrostomy and cystostomy catheters were passed through the body wall, paramedian to the abdominal incision on the ipsilateral side of the affected kidney, and connected to a shunting port. For the SUB 3.0, the nephrostomy and cystostomy catheters were connected to either a Y‐connector, if the SUB was unilateral or an X‐connector if bilateral (Figure S2b,c). In some cases, bilateral obstructions had a unilateral 2.0 and 3.0 device, and in other cases a bilateral obstruction had two Y‐connectors with two separate SUB 2.0 or 3.0 systems. This decision was clinician dependent.

### 2.5 Perioperative Data and Management

The cats were monitored for hydration using a combination of physical examination, serial bodyweight measurements, and point‐of‐care bloodwork. Antibiotic therapy (marbofloxacin ~2.75 mg/kg once daily PO via esophagostomy tube) was initiated to prevent the formation of biofilm on the internalized device for 2 weeks unless the cat was receiving another antibiotic for a known UTI [22]. If a positive culture was obtained, an appropriate antibiotic was given for ~6 weeks. Electrolytes, BUN, creatinine, packed cell volume/total solids (PCV/TS) were recorded postoperatively and then at least every 24 h after surgery until discharge. The SUB was flushed before discharge.

For each SUB flush (routinely done without sedation), the cat was placed in dorsal recumbency and the port site on the ventral abdomen was clipped of fur and aseptically prepared as defined in the instruction for use manual (Figure S1).^4^ Ultrasonography was used to measure the renal pelvic/ureteral diameter, evaluate the lumen of the urinary bladder and entry site of the cystostomy catheter, and assess the catheter tubing for shadowing or mineralization. A sterile 22G Huber needle connected to a T‐port and three‐way stopcock were used to obtain a sterile urine sample from the port. For SUB version 3.0, a 0.5 mL presample was removed and discarded before obtaining a sample for culture, as the accessory line could have stagnant tEDTA solution from the prior flush. Sterile saline was then infused using ultrasound‐guidance in 0.25–0.5 mL increments and removed after each infusion, while the renal pelvis and the urinary bladder were monitored serially with ultrasonography for turbulence of fluid to confirm device patency. Once patency was established, 2 mL of 2% tEDTA was slowly infused in 0.25 mL increments as the renal pelvis was carefully visualized with ultrasonography to avoid overdistension.

### 2.6 Follow‐Up

Typically, evaluation of renal variables, urinalysis/UCS, and a urinary ultrasonography was performed 4 weeks postoperatively, and then every 3 months for the first 2 years, and every 6 months thereafter. Flush interval was calculated as the average number of days between flushes for each device.

Mineralization was defined as any occurrence where the device had ultrasonographic shadowing debris appreciated on the catheter(s), while the catheters were flowing and draining. In addition, mineralization was presumptively diagnosed if there were any delay in the visualization of turbulence during the SUB flush, even if flow was documented, if there was an absence of other causes or poor flushing (e.g., kink, blood clot, purulent debris). Complete occlusion was characterized when one or more catheters were unable to be flushed. Exchange was recommended if there was occlusion of any part of the device with associated renal pelvic/ureteral dilation supporting recurrent obstruction. If there was occlusion of the device without evidence of progressive renal pelvic/ureteral dilation, the renal collection system was assumed to be patent, likely associated with resolution of a previous obstruction in the ureteral lumen. In these cases, exchange of the device was not recommended.

### 2.7 Statistical Analysis

Descriptive statistics are presented as means and a standard deviation. Mean differences were carried out by ANOVA. Regression analyses were carried out using OLS for continuous response variables while discrete response variables were analyzed using logistic regression. Frequency analyses were carried out using a Pearson's chi‐square or Fisher's exact test for cell counts less than five. All analyses were carried out using SAS 9.4 (Cary, NC 2016).

## 3 Results

### 3.1 Selection of Cases (Table 1)

**TABLE 1:** Cats and devices organized by Group: Number of cats and devices in each group based on laterality, follow‐up times, and comparisons of median follow‐up times between groups.

	Cats	Devices	Unilateral	Bilateral	Median follow‐up time (mean, range) in days
Group 1	*n* = 21	*n* = 28	*n* = 14	*n* = 7	772 (751.5, 194–1345)
(compared to Group 2)					*p* = 0.340
Group 2	*n* = 12	*n* = 16	*n* = 8	*n* = 4	934.5 (978.4, 335–2367)
(compared to Group 3)					*p* = 0.572
Group 3	*n* = 33	*n* = 51	*n* = 17	*n* = 17	1015.5 (1067.5, 183–2393)
	(SUB 2.0: 17 cats)				
(compared to Group 1)
(SUB 3.0: 16 cats)				*p* = 0.346

Records for 349 devices in 254 cats were reviewed between 2012 and 2020. Ninety‐five devices (39 unilateral, 28 bilateral) in 66 cats met the inclusion criteria with 28 devices (14 unilateral, 7 bilateral) in 21 cats in Group 1, 16 (8 unilateral, 4 bilateral) in 12 cats in Group 2, and 51 (17 unilateral, 17 bilateral) in 33 cats in Group 3.

### 3.2 Historical and Presenting Clinical Data

Median age at the time of the procedure was 10.9 years (range: 0.6–17.2). Age was significantly different between Groups 1 and 2 (mean: 13.2 and 10, respectively; *p* = 0.0013), and between Groups 1 and 3 (mean: 8.6; *p* < 0.001). There was no significant difference in age between Group 2 and 3 (*p* = 0.21). Forty of 66 (61%) cats had a history of azotemia and were suspected to have CKD based on small kidney size, a history of polyuria and polydipsia, a loss of corticomedullary definition on ultrasonography, or a combination; there was no significant difference between groups. Eight of 66 (12%) cats had previously undergone cystotomy for removal of cystic calculi and there was no significant difference between groups. Nine of 66 (14%) cats had a history of previous UTI before SUB placement and there was no significant difference between Groups 1 and 2 (*p* = 0.26), 1 and 3 (*p* = 0.24), and 2 and 3 (*p* = 0.29). Three of 66 (5%) cats experienced pre‐procedure dysuria; in two cats this was thought to be idiopathic cystitis and in the third it was secondary to a urethral stone.

### 3.3 Clinicopathologic and Laboratory Data

Overall, the median creatinine at time of surgery was 8.35 mg/dL (range: 1.3–24.8; reference: 0.9–2.3). Median creatinine was 8.2 mg/dL (mean: 9.2, range: 1.7–23) for Group 1, 7.7 mg/dL (mean: 7.8, range: 1.7–17.6) for Group 2, and 8.7 mg/dL (mean: 9.5, range: 1.3–24.8) for Group 3. There was no significant difference in creatinine between groups.

Overall, the median ionized calcium at time of surgery was 1.05 mmol/dL (range: 0.75–1.68, reference: 1.20–1.32). Sixteen of 18 (88%) cats in Group 1, 12/12 (100%) cats in Group 2, and 28/30 (93%) cats in Group 3 had a normal ionized calcium before surgery; 1 cat in Group 1 had a low ionized calcium and 3 cats (1, Group 1; 2, Group 3) had an elevated ionized calcium before surgery. There was no significant difference in incidence of preoperative hypocalcemia, normocalcemia, or hypercalcemia between groups.

Seven of 66 (11%) cats (3/21 [14%] in Group 1, 1/12 [8%] in Group 2, 3/33 [9%] in Group 3) had a positive urine culture at the time of surgery. There was no significant difference between the groups. Forty‐two of 66 (67%) cats had a UCS performed from a sample obtained via pyelocentesis. Four of 42 (10%) cats (1/13 [8%] in Group 1, 1/5 [20%] in Group 2, 2/24 [8%] in Group 3) had a positive UCS from the pelvis. There was no significant difference in a positive culture obtained via pyelocentesis between groups. In two cats, culture obtained via cystocentesis was positive while the culture obtained via pyelocentesis was negative. No cats that had a negative culture via cystocentesis had a positive culture via pyelocentesis. Of the seven cats with positive urine cultures, all were on antibiotic therapy for at least 72‐h before to surgery. There was no significant difference in preoperative antibiotic therapy between groups.

### 3.4 Diagnostic Imaging

The median preoperative renal pelvic diameter measurements obtained with ultrasonography in a transverse plane was 9 mm (range: 1–35 mm). Renal pelvic diameter was significantly different (*p* = 0.016) between Groups 2 (mean: 12.2 mm) and 3 (mean: 7.8 mm), but not significantly different between Groups 1 (mean: 9.9 mm) and 2 or 3.

The etiology for obstruction included ureterolithiasis (74/95; 78%), presumed stricture (21/95; 22%), and purulent debris associated with pyonephrosis (6/95; 6%). Eleven of 74 (15%) ureterolith cases were suspected to be obstructed with dried solidified blood stones based on gross appearance of the stones intraoperatively and 3/74 ureters had concurrent strictures, totaling 24/95 (25%) cats with presumed strictures in this study. There was no significant difference between groups for incidence of ureterolithiasis, stricture, or purulent debris.

### 3.5 SUB Placement Procedure

SUBs were successfully placed in all cases. For 79/95 (83%) obstructed ureters SUB 2.0 devices were placed and for 16/95 (17%) obstructed ureters SUB 3.0 devices were placed. All SUB 3.0s were in Group 3. Two SUB 2.0s in Group 3 were exchanged for a SUB 3.0 at 103 and 2113 days, respectively, following the initial surgery for complete occlusion secondary to mineralization.

An intraoperative cystotomy was performed in 14/66 (21%) of cats for concurrent cystolithiasis; there was no significant difference between groups for the need for a cystotomy. Five cats in Group 1, but no cats in Group 2 or 3 had an indwelling urinary catheter placed postoperatively to monitor urine output.

### 3.6 Follow‐Up (Table 1)

The median survival time was 772 days (range: 194–1345; mean: 751.5) for Group 1, and 934.5 days (range: 355–2367; mean: 978.4) for Group 2 with all cats documented dead at the time of writing this manuscript. There was no significant difference between groups. In Group 3, 55% of cats were alive at the time of writing this manuscript, therefore, median survival times could not be calculated, but the median follow‐up time was 1015.5 days (range: 183–2393; mean: 1067.5), which was not statistically different than the MST of Groups 1 or 2. No cats in this study were lost to follow‐up. There was no significant difference in follow‐up time between groups.

### 3.7 Renal Variables (Table 2)

**TABLE 2:** Follow‐up and Group comparisions of creatinine concentrations of cats after SUB placement.

	1 month	3 months	6 months	9 months	12 months
Group 1, *n* = 21	*n* = 21	*n* = 19	*n* = 19	*n* = 15	*n* = 15
Mean (mg/dL)	2.9	2.85	3.24	3.3	3.8
Range	1.5–5.1	1.4–4.8	1.3–5.5	1.6–6.1	1.5–10.3
(compared to Group 2)	*p* = 0.836	*p* = 0.096	*p* = 0.351	*p* = 0.466	*p* = 0.07
Group 2, *n* = 12	*n* = 12	*n* = 12	*n* = 9	*n* = 9	*n* = 11
Mean (mg/dL)	3.0	2.3	2.8	2.9	2.6
Range	1.5–10.8	1.4–4.1	1.2–4.3	1.3–5	1.4–5.2
(compared to Group 3)	*p* = 0.785	*p* = 0.091	*p* = 0.966	*p* = 0.608	*p* = 0.877
Group 3, *n* = 33	*n* = 32	*n* = 33	*n* = 31	*n* = 27	*n* = 27
Mean (mg/dL)	2.8	2.9	2.8	2.6	2.5
Range	0.9–7.4	1.1–7.3	1.1–7	1.2–6.7	1.3–3.9
(compared to Group 1)	*p* = 0.876	*p* = 0.813	*p* = 0.287	*p* = 0.082	*p* = **0.042**

*Note:* Bolding of data = significant difference with *p* < 0.05.

Creatinine concentrations at 1, 3, 6, 9, and 12 months postoperatively were compared and are shown in Table 2. At 1, 3, 6, and 9‐month postoperatively there was no significant difference in creatinine between Groups 1, 2, and 3. At 12 months postoperatively, mean creatinine was 3.8 mg/dL (range: 1.5–10.3) for Group 1 (*n* = 15), 2.6 mg/dL (range: 1.4–5.2) for Group 2 (*n* = 10), and 2.5 mg/dL (range: 1.3–3.9) for Group 3 (*n* = 27). There was a significant difference at 12 months between Groups 1 and 3 (*p* = 0.042), but not between Groups 1 and 2 (*p* = 0.07) or Groups 2 and 3 (*p* = 0.877).

### 3.8 Mineralization (Tables 3 and 4)

**TABLE 3:** Group comparisons of device mineralization, occlusion, and need for device exchange.

	Mineralization	Occlusion	Exchange
Group 1	9/28	8/28	4/28
	32%	29%	14%
(compared to Group 2)	*p* = 0.337	*p* = 0.738	*p* = 0.250
Group 2	8/16	6/16	5/16
	50%	38%	31%
(compared to Group 3)	*p* = **0.025**	*p* = 0.065	*p* = **0.016**
Group 3	10/51	7/51	3/51
	19%	14%	6%
(compared to Group 1)	*p* = 0.273	*p* = 0.137	*p* = 0.237

*Note:* Bolded *p*‐values are considered statistically significant.

**TABLE 4:** Group comparisons of median time to mineralization occlusion and first UTI.

	Occlusion	1st UTI
Group 1	8/28 devices (29%)	6/21 cats (29%)
Median (days)	438.5	104
Mean (days)	521	235.7
Range (days)	320–793	6–1009
(compared to Group 2)	*p* = 0.523	*p* = 0.360
Group 2	7/16 devices (38%)	5/12 cats (42%)
Median (days)	455	251
Mean (days)	472	358
Range (days)	238–1073	6–963
(compared to Group 3)	*p* = 0.701	*p* = 1.000
Group 3	7/51 devices (14%)	5/33 cats (15%)
Median (days)	480	222
Mean (days)	588	316
Range (days)	70–2113	8–635
(compared to Group 1)	*p* = 0.772	*p* = 0.523

Evidence of mineralization of the device on ultrasound occurred in 9/28 (32%), 8/16 (50%), and 10/51 (19%) in Groups 1, 2, and 3, respectively. There was a significant difference between Groups 2 and 3 (*p* = 0.025). Complete occlusion of the SUB from mineralization occurred in 8/28 (29%), 6/16 (38%), and 7/51 (14%) devices in Groups 1, 2, and 3, respectively, with no significant difference between groups. Device exchange was needed in 4/28 (14%), 5/16 (31%), and 3/51 (6%) devices in Groups 1, 2, and 3, respectively. There was a significant difference between Groups 2 and 3 (*p* = 0.016), but not between Groups 1 and 2 (*p* = 0.25) or Groups 1 and 3 (*p* = 0.24). In two of four (50%) Group 1 device exchanges and four of five (80%) Group 2 device exchanges, more than one device exchange was needed due to recurrence of complete occlusion from mineralization. None of the exchanged devices in Group 3 were exchanged more than once at the time of last follow‐up. Of the 17 SUB 3.0 devices in Group 3 there was no significant difference in rate of mineralization compared to SUB 2.0 devices in Group 3 but a majority of the devices were the 2.0 version.

The median time to complete occlusion (Table 4) from mineralization was 438.5 days (range: 320–793; mean: 521) for Group 1, 455 days (range: 238–1073; mean: 472) for Group 2, and 480 days (range: 70–2113; mean: 588) for Group 3. There was no significant difference between groups for time to mineralization occlusion.

When the cause of obstruction was considered, cats with ureterolithiasis as a cause had a median time to mineralization occlusion of 488 days (range: 238–1073; mean: 531.1), while those with non‐ureteroliths as a cause were 339 days (range: 238–541; mean: 364.5) (*p* = 0.16). There was no significant association between those with ureterolithasis and mineralization (*p* = 0.33), time to mineralization (*p* = 0.25), complete occlusion from mineralization (*p* = 0.99), or need for SUB exchange (*p* = 0.19).

Postoperative hypercalcemia was documented in 3/21 (14%), 2/12 (16%), and 5/33 (15%) of cats in Groups 1, 2, and 3, respectively; there was no significant difference between groups. There was no association between postoperative hypercalcemia and mineralization (*p* = 0.08), time to documentation of mineralization (*p* = 0.16), complete occlusion from mineralization (*p* = 0.37), time to complete occlusion from mineralization (*p* = 0.37), and need for SUB exchange from mineralization (*p* = 0.08), though it was approaching significance at *p* = 0.08 for an association of hypercalcemia and mineralization. Of the 10 cats that had documentation of hypercalcemia, 7/10 cats were medically managed for idiopathic hypercalcemia (based on PTH profile), given medications to try and minimize calciuria, or both. Therefore, these cats were managed for their hypercalcemia during the follow‐up period. Treatment generally consisted of alendronate (10 mg/week) and oral hydrochlorothiazide (~1 mg/kg q12h PO), potassium citrate (100–150 mg/kg/day PO), or both. Follow‐up ionized calcium levels were not available for review in all cats to report medical success.

### 3.9 Infection (Tables 4 and 5)

**TABLE 5:** Group comparisons of data on cats for infection by group.

	Any bacteriuria	Chronic UTI	Cleared infection
Group 1	7/21	7/21	0/7
	33%	33%	0%
(compared to Group 2	*p* = 0.347	*p* = 1.000	*p* = 0.192
(compared to Group 2	6/12	4/12	2/6
	50%	33%	33%
Group 3	*p* = 0.147	*p* = **0.014**	*p* = 0.091
Group 3	8/33	1/33	7/8
	24%	3%	88%
(compared to Group 1)	*p* = 0.541	*p* = **0.004**	*p* = **0.001**

*Note:* Bolding of data = significant difference with *p* < 0.05.

Documentation of any postoperative bacteriuria occurred in 7/21 (33%), 6/12 (50%), and 8/33 (24%) of cats in Groups 1, 2, and 3, respectively, which were not statistically different. Chronic UTI, as defined above, was associated with clinical UTIs and occurred in 7/21 (33%), 4/12 (33%), and 1/33 (3%) cats in Groups 1, 2, and 3, respectively. There was a significant difference between Groups 1 and 3 (*p* = 0.004) and Groups 2 and 3 (*p* = 0.014), but not between Groups 1 and 2 (*p* = 1.0). Four of 12 cats (33%) with chronic UTIs (1/7 Group 1, 2/4 Group 2, and 1/1 Group 3) were actively infected at the time of surgery. Seven of 12 cats (58%) with chronic UTI (3/7 [43%] Group 1, 3/4 [75%] Group 2, and 1/1 [100%] Group 3) had a history of chronic infections before SUB placement.

UTI at the time of surgery was significantly (*p* = 0.01) associated with the development of a chronic UTI. Infections were cleared in zero of seven (0%), two of six (33%), and seven of eight (88%) cats in Groups 1, 2, and 3, respectively, in cats that had one or more UTIs throughout the study. There was a significant difference between Groups 1 and 3 (*p* = 0.001). The median time to first UTI was 104 days (range: 6–1009; mean: 235.7) for Group 1, 251 days (range: 6–963; mean: 358) for Group 2, and 222 days (range: 8–635; mean: 316) for Group 3 and no group was statistically different.

### 3.10 Dysuria

Four of 66 (6%) cats had postoperative dysuria (1, Group 2; 3 Group 3). Three of the four cats had preoperative dysuria. Two of these three cats had idiopathic cystitis and continued to have intermittent episodes of dysuria postoperatively. One of the cats with preoperative dysuria had a urethral stone preoperatively and developed postoperative dysuria with a fibrous band of tissue that formed around the bladder body distorting the shape; the dysuria resolved following removal of that band and the cystostomy catheter. This cat had resolution of the ureteral obstruction based on ureterogram and the SUB was removed from the cats urinary bladder at the time of the second surgery. The remaining cat with a newly developed postoperative dysuria was thought to have idiopathic cystitis given the lack of temporal association with tEDTA infusion, absence of bacteriuria, the intermittent signs associated with stress, and the self‐limiting nature. There was not a significant difference in the incidence of dysuria in cats that received a SUB 3.0 or 2.0 device in Group 3.

## 4 Discussion

The goal of this study was not to compare SUB 2.0 and 3.0 devices and that work is ongoing by the authors. The results of this study suggest that the prophylactic use of tEDTA might be helpful in decreasing SUB mineralization rates and postoperative bacteriuria and associated UTIs, without reducing postoperative renal function or causing dysuria. There were fewer cats in this study that received a SUB 3.0 device compared to a 2.0, but there was no detectible increase in complications based on device model and the rate of complications in this study. Aside from the mean and median age of cats in Groups 1 and 3 being significantly different, over half of the cats in Group 3 were still alive at the time of data collection. The median survival/follow‐up times were not significantly different for Group 3 cats (1116 days), compared to Groups 1 (772 days) and 2 (934 days), supporting the duration of the time the implant was indwelling was comparable between groups.

SUB device mineralization, occlusion from mineralization, or the need for device exchange occurred less often in Group 3 devices (19%, 14%, 6%, respectively) compared to Group 1 (32%, 29%, 14%, respectively) or Group 2 devices (50%, 38%, 31%, respectively). The rate between Groups 2 and 3 were statistically different for mineralization (*p* = 0.025) and exchange (*p* = 0.016).

No group had a statistically different mean or median time to first occlusion, but Group 3, which had the longest mean and median follow‐up time overall, had a longer mean time to mineralization occlusion, and had less occlusions reported overall (29%, 38%, and 14% in Groups 1, 2, and 3, respectively). Additionally, of the devices exchanged, 50% of those in Group 1 and 80% of those in Group 2 required more than one exchange per device, while those in Group 3 only required one. It is important to consider all groups having similar follow‐up times that go beyond the time of median device mineralization that has been previously reported (463 days) to ensure the data is comparable covering a time period where the event is expected to occur.

The development of postoperative hypercalcemia was also evaluated to assess for a potential confounding factor causing mineralization, but there was no significant difference in incidence between groups despite there being a difference in mineralization rates. One additional factor to consider is that over time the authors' have become more aggressive in the treatment of marginal hypercalcemia with hydrochlorothiazide, potassium citrate, high fiber diets, chia seed intake, and bisphosphonate therapy, when indicated. This could account for the fact that cats that have evidence of hypercalcemia have improvements over time and are on chelators to minimize calcium in the urine. Therefore, they might have less mineralization due to that aggressive therapy. Despite that, a minority of the cats in each group were hypercalcemic.

Evidence of a chronic UTI occurred less often in Group 3 cats (3%) compared to Group 1 (33%) and Group 2 (33%). Additionally, cats in Group 3 were more often able to clear their bacteriuria long‐term (88% compared to 0% in Group 1 and 33% in Group 2). There were significant differences between Groups 1 and 3 for incidence of chronic UTI (*p* = 0.004) and ability to their clear infection (*p* = 0.001). While there was no significant difference between Groups 1 and 3 for incidence of any bacteriuria developing (33% vs. 24%, respectively) the development of chronic UTIs was statistically significantly less for Group 3 (33% vs. 3%, respectively). These findings suggest that compared to sterile saline, tEDTA might be protective long‐term against the development of chronic infections and preventative for recurrent infections. In addition, if bacteriuria was documented at the time of surgery based on a positive urine culture, there was a statistical association with that cat developing chronic UTIs in the long‐term (*p* = 0.01), which has been documented previously [1].

Another interesting finding was that no cat had a positive culture by pyelocentesis without a positive culture by a cystocentesis, whereas two cats had a positive culture by cystocentesis and not by pyelocentesis, supporting that occult pyelonephritis in cats might not be a common occurrence and doing diagnostic pyelocentesis in cats that have a negative urine culture by cystocentesis to screen for pyelonephritis may not be warranted.

Renal variables at multiple time points during the follow‐up period were not significantly different between groups except for creatinine concentrations at 12 months postoperatively between Groups 1 and 3 (*p* = 0.042), where Group 3 had a lower mean creatinine (2.5 mg/dL) compared to Group 1 (3.8 mg/dL). This suggests that tEDTA infusions do not exacerbate chronic azotemia. The relative differences between Groups 1 and 3 could be due to several factors including greater age in Group 1, improved medical care in recent years, longer follow‐up times with a larger number of cats followed long‐term in Group 3, or improved outcomes due to a decreased incidence of mineralization and infection causing additional renal insult.

There are several limitations of this study. Many cats that would have been in Group 1 were excluded due to an incomplete medical record or a lack of documentation of routine SUB flushing because at the time of the first SUB placement, routine flushing was not the standard of care and there were no electronic medical records for the authors to capture this data. To consistently compare groups, the authors were careful to ensure that all medical records were complete, at least 180 days of follow‐up time was available, so all groups were comparable to known historical incidence of complications, and the full records were available for review, as well as similar follow‐up details on each cat. Overall, this limited the number of cats in each group meeting the inclusion criteria. Additionally, the degree of follow‐up needed to meet inclusion criteria could have created a bias population as perhaps cats that experienced mineralization or UTI had owners that were more likely to seek care and adhere to the recommendations for reevaluation compared to cats that did not experience these issues, or the opposite could be true and those cats that followed a rigorous follow‐up schedule were more likely to be maintained on quality diets, managing their hypercalemia more aggressively, and more strictly returning for SUB flush maintenance. Group 2 was a difficult group to analyze because some cats could have spent 3 years being flushed with saline and 6 months with tEDTA, and others 3 months with saline and years with tEDTA, pending the year of their surgery. Both circumstances would have fit within the inclusion criteria for Group 2 and not Groups 1 or 3. We felt this group was important to evaluate to see if there was a trend in that group away from Group 1 and toward Group 3 or vice versa. Due to the small numbers this resulted in the data from this group being harder to interpret.

Additionally, Group 3 cats started having the SUB 3.0 device placed in 2019, toward the end of this collection period. It was hard to determine if any of the differences were due to the 3.0 version of the device compared to the 2.0 version of the device but the data evaluated did not suggest there was a difference. The cats with the 3.0 device had shorter follow‐up times than those with a 2.0 device, naturally. Finally, Group 3 cats had the longest follow‐up times compared to the other groups with a majority of cats still being alive. This could have biased the study sample to lead to higher mineralization or infection rates overtime, but instead they had lower rates compared to the two other groups, supporting the hypothesis even further that tEDTA could support decreasing these two long‐term complications and should be considered in the long‐term management of these devices. One additional limitation was the failure to include a group of cats over the same long‐term period of time that did not have any flushing performed. Finally, over the time course of this study medical management for CKD, hypercalcemia and SUB flushing/exchange could have become more aggressive, biasing the two populations between Groups 1 and 3. The protocols for medical management and SUB maintenance over this time period, in the authors' practice, are identical. The only modification was the addition of tEDTA to the flushing regimen, making this potential unlikely to confound the results.

In conclusion, prophylactic use of 2% tEDTA could be helpful at decreasing mineralization, the need for device exchange, chronic postoperative urinary tract infections, and the ability to clear infections in cats after SUB placement for BUOs. tEDTA did not result in any obvious adverse effects on renal function or the development of dysuria. The authors consider infusion of tEDTA part of their standard‐of‐care in managing cats with the SUB device(s) long‐term.

## Disclosure

Authors declare no off‐label use of antimicrobials.

## Ethics Statement

Authors declare no Institutional Animal Care and Use Committee (IACUC) or other approval was needed. Authors declare human ethics approval was not needed.

## Conflicts of Interest

Drs Berent and Weisse are consultants for Norfolk Vet and Infiniti Medical Vet Products and designed the SUB device.

## Supporting information


**Data S1.** Supporting Information.

## Endnotes

^1^
Tetrasodium EDTA, Norfolk Vet Products, Skokie, IL.

^2^
SUB device, Norfolk Vet Products, Skokie, IL.

^3^
SUB device instruction for use manual (https://norfolkvetproducts.com/wp‐content/uploads/2020/10/SUB3_Surgical_Guide_2020‐09‐email.pdf).

^4^
SUB flush instruction for use manual (https://norfolkvetproducts.com/PDF/SUB/SUB_Flush_Guide_2018‐01‐email.pdf).
