# Supplementary material for: Long‐Term Outcomes After Prophylactic Infusion of 2% Tetrasodium Ethylenediaminetetraacetic Acid in 95 Subcutaneous Ureteral Bypass Devices in 66 Cats With Benign Ureteral Obstructions
Source: J Vet Intern Med. 2025 Feb 26;39(2):e70006. doi: 10.1111/jvim.70006 (PMC11864850; doi:10.1111/jvim.70006)

Supplemental Figure 1: Ultrasound-guided flushing of a SUB device with cat in dorsal recumbency and the fur is clipped with the skin ascetically prepared. A) Huber needle is placed into the subcutaneous access port of the SUB (black arrow) and it Is connected to a t-port (red arrow), 3 way stop-cock (yellow arrow) and 2 syringes; one for urine to discard (white arrow) and one for urine to analyze (blue arrow). B) ultrasonography being used to assess the kidney and urinary bladder. Urine collected for analysis (blue arrow). C) Renal ultrasonography during sub flushing with sterile saline (white arrow). D) ultrasonography of the kidney during flushing as 2 mL of tEDTA is instilled into the device slowly.

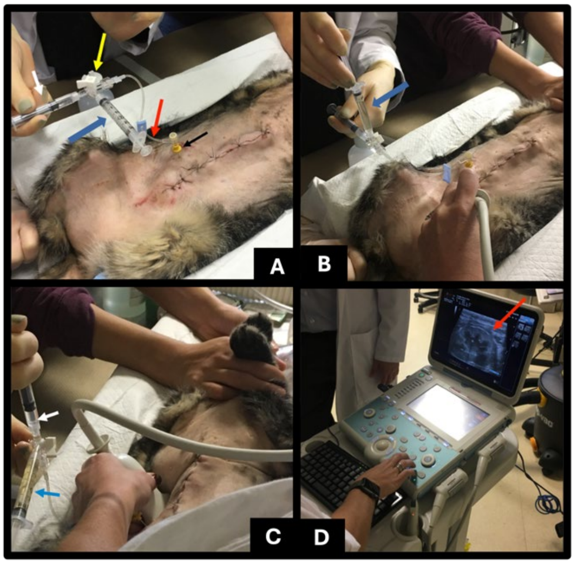

Supplemental Figure 2: Fluoroscopic and schematic images of the SUB 2.0 and 3.0 devices. A) Locking loop nephrostomy and cystostomy tube of the SUB 2.0. B) Fluoroscopy image of the SUB 2.0 in a cat. C) Ventrodorsal fluoroscopic image of a left sided unilateral SUB 3.0 device. D) Ventrodorsal fluoroscopic image of a right sided unilateral SUB 3.0 device. E) Schematic of the unilateral SUB 3.0 device. F) Fluoroscopic image of a bilateral SUB 3.0 device in ventrodorsal positioning. G) Lateral fluoroscopic image of a bilateral SUB 3.0 device. H) Schematic of a bilateral SUB 3.0 device.

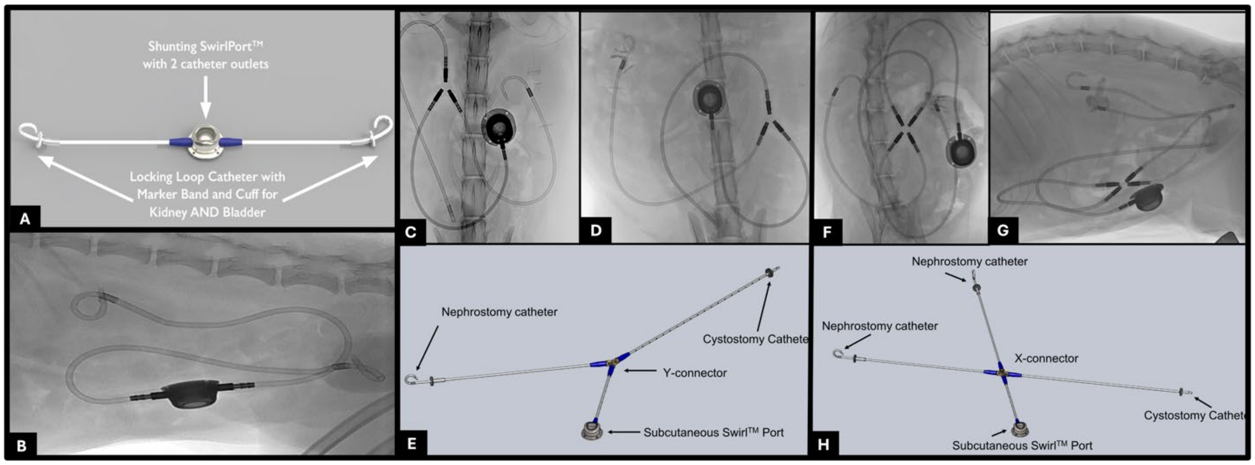

Supplement: Supplementary file 1 — Data S1. Supporting Information. [file JVIM-39-e70006-s001.pdf]
